# Efficacy and Tolerability of Fixed-Dose Combination of Dexketoprofen and Dicyclomine Injection in Acute Renal Colic

**DOI:** 10.1155/2012/295926

**Published:** 2012-04-23

**Authors:** A. Porwal, A. D. Mahajan, D. S. Oswal, S. S. Erram, D. N. Sheth, S. Balamurugan, V. Kamat, R. P. Enadle, A. Badadare, S. K. Bhatnagar, R. S. Walvekar, S. Dhorepatil, R. C. Naik, I. Basu, S. N. Kshirsagar, J. V. Keny, S. Sengupta

**Affiliations:** ^1^Sharada Clinic, 408/1, Shankar Sheth Road, near Ekbote Colony, Ghorpade Peth Pune 411042, India; ^2^Sai Urology Hospital, Plot No. 1, Vishal Nagar, Gajanan Mandir Road, Aurangabad 431005, India; ^3^Modern Stone Care & Urology Research Centre, 434/10 Saraja Vaibhav, 1st Foor, Opp. Tathe Hospital, Shaniwar Peth, Karad 415110, India; ^4^Sharada Clinic, Erram Hospital, near Krishna Bridge, Station Road, Karad 415110, India; ^5^Surgical Hospital, Opp Municipal School, Nr. Vishvakunj Society, Narayan Nagar Road, Paldi, Ahmedabad 380007, India; ^6^Chest Research Centre, 2, Janki Nagar Extension, Valasaravakkam, Chennai 600087, India; ^7^Department of Surgery, Karnataka Institute of Medical Sciences, Hubli 580022, India; ^8^Prabhavati Multi Speciality Hospital and Research Centre, Ambejogai Road, Latur 413512, India; ^9^Giridhar Clinic, Oshiya Corner Building, Sukhsagar Nagar, Pune 411046, India; ^10^Abhinav Multispeciality Hospital, Kamal Chowk, Naya Nakasha, Nagpur 440017, India; ^11^Walvekar Hospital, Opp Garpir, 6th lane, Ganesh Nagar, Sangli 416416, India; ^12^Shree Hospital, Siddharth Mansion, Nagar Road, Pune 411014, India; ^13^Ketki Hospital, Plot No. 477, N-3, CIDCO, near Kamgar Chowk, Opp. to Chate House, Aurangabad 431001, India; ^14^Ramkrishna Mission Hospital, Luxa, Varanasi 221010, India; ^15^Sevadham Hospital, Talegaon Dabhade, Talegaon Dabhade Station, Pune 410506, India; ^16^Prabha Vithal Clinic, 166/ FNM Shivaji Nagar, Sion Agaarwada Road, Sion (East), Mumbai 400022, India; ^17^Sengupta Hospital, Research Institute, Ravi Nagar, Nagpur 440033, India

## Abstract

*Objective.* To evaluate the efficacy and tolerability of a fixed-dose combination of dexketoprofen and dicyclomine (DXD) injection in patients with acute renal colic. *Patients and Methods.* Two hundred and seventeen patients were randomized to receive either DXD (*n* = 109) or fixed-dose combination of diclofenac and dicyclomine injection (DLD; *n* = 108), intramuscularly. Pain intensity (PI) was self-evaluated by patients on visual analogue scale (VAS) at baseline and at 1, 2, 4, 6, and 8 hours. Efficacy parameters were proportion of responders, difference in PI (PID) at 8 hours, and sum of analogue of pain intensity differences (SAPID). Tolerability was assessed by patients and physicians. *Results.* DXD showed superior efficacy in terms of proportion of responders (98.17% versus 81.48; *P* < 0.0001), PID at 8 hours (*P* = 0.002), and SAPID_0–8 hours_ (*P* = 0.004). The clinical global impression for change in pain was significantly better for DXD than DLD. The incidence of adverse events was comparable in both groups. However, global assessment of tolerability was rated significantly better for DXD. *Conclusion.* DXD showed superior efficacy and tolerability than DLD in patients clinically diagnosed to be suffering from acute renal colic.

## 1. Introduction

Acute renal colic (ARC) is a common emergency condition mimicking acute abdominal or pelvic condition. About 12% of the population is likely to suffer from ureteric colic sometime in their lifetime and recurrence rates can approach about 50% [1]. It is extremely important to relieve the excruciating pain associated with this condition and establish a confirmatory radiological diagnosis at the earliest onset.

The severe pain of ARC is due to increasing wall tension in the urinary tract as a result of obstruction of the urinary flow. The rising pressure in renal pelvis stimulates release of prostaglandins that cause vasodilatation. This leads to diuresis and thus further increase in the intrapelvic pressure. Prostaglandins also lead to ureteric spasm that further amounts to pain [2, 3].

Parenteral nonsteroidal anti-inflammatory drugs (NSAIDs) have been used widely for the treatment of ARC and have been shown to achieve greater reduction in pain scores than opioids. The use of NSAIDs has reduced the requirement for further analgesia beyond short term [4]. Unlike opioids, NSAIDs not just symptomatically relieve pain but also inhibit synthesis of prostaglandins, which are involved in the etiopathogenesis.

Spasmolytics are traditionally used in renal colic, biliary colic, or dysmenorrhoea for relief of smooth muscle spasm. As spasmolytics relieve the pain associated with smooth muscle spasm, the combination of NSAIDs with spasmolytics is likely to be synergistic. Fixed dose of combinations (FDC) of mefenamic acid, aceclofenac with spasmolytics such as dicyclomine or drotaverine have been demonstrated to be highly effective in relief of acute spasmodic pain [5, 6]. In the study performed by Pareek et al., addition of spasmolytic such as drotaverine to aceclofenac was found to provide significant therapeutic benefit as compared to monotherapy with aceclofenac [6].

The parenteral formulation of dexketoprofen trometamol, the S-enantiomer of ketoprofen, has shown good safety and efficacy in the treatment of ARC in previous studies [3, 7]. The present study was planned to evaluate the efficacy and tolerability of FDC of an NSAID, dexketoprofen with dicyclomine (DXD) injection in the treatment of clinically diagnosed ARC when administered as an intramuscular (IM) injection. To our knowledge, this is the first clinical study reported for this FDC.

## 2. Patients and Methods

### 2.1. Objective

The objective of this study was to compare the efficacy and tolerability of FDC of dexketoprofen and dicyclomine IM injection (DXD) with FDC of diclofenac and dicyclomine IM injection (DLD) in the treatment of patients clinically diagnosed to be suffering from ARC.

### 2.2. Study Design

This was a randomised controlled, multicentric, open-label, parallel group study conducted at different centres across India. The study was approved by institutional review board or independent ethics committee for each centre. Written informed consent was provided by each participant prior to any study-related procedure. The execution and monitoring of the study were done in accordance with the requirements of Good Clinical Practice.

### 2.3. Study Population

The study population involved male and female patients between 18 and 65 years of age presenting with acute colicky pain in the flank and/or radiating to the abdomen or genitalia. Patients with moderate to severe pain on visual analogue scale (VAS ≥40 mm) and willing to provide written informed consent were included in the study. The important exclusion criteria included hypersensitivity to the study medications or intolerance to NSAIDs or any anesthetic medication; active or suspected gastrointestinal ulcer, chronic dyspepsia, or gastrointestinal bleeding; Crohn's disease or ulcerative colitis; history of bronchial asthma, severe heart failure/moderate-to-severe renal dysfunction (creatinine clearance <50 mL/min.), or severely impaired hepatic function (Child-Pugh score 10–15); hemorrhagic diathesis and other coagulation disorders; contraindication to use of NSAIDs; diagnosed gastrointestinal obstruction; myasthenia gravis; glaucoma.

### 2.4. Treatment Procedure

Patients presenting with acute colicky pain in the flank region were screened based on complete medical history and examination. Patients satisfying the selection criteria were randomised to receive either FDC of dexketoprofen (as trometamol) 50 mg and dicyclomine 20 mg IM injection (DXD) [manufactured by Emcure Pharmaceuticals Ltd., Pune] or FDC of diclofenac (as sodium) 50 mg and dicyclomine 20 mg IM injection (DLD) [from commercial source]. Patients were randomized in 1 : 1 ratio to “DXD” or “DLD” using blocks of 10 through online randomization software available at http://www.randomization.com/. Any concomitant therapy deemed necessary was provided for the patients as per investigator's discretion. However, any other analgesic, anti-inflammatory, or muscle-relaxant therapy, and products from alternative system of medicine with analgesic, anti-inflammatory action were not allowed. The patients were simultaneously investigated radiologically for renal pathology.

### 2.5. Efficacy Variables

The intensity of pain was assessed from VAS at baseline and at the end of 1, 2, 4, 6, and 8 hours after administration of study medication. At least 50% improvement in pain score at 8 hours was considered as the responder's criterion. Proportion of responders in each study group was considered as primary efficacy variable.

The secondary variables included pain intensity difference (PID) after 8 hours of injection and sum analogue of pain intensity difference (SAPID) over 8 hours.

PID was calculated for each observation by subtracting the present PI from the baseline value. SAPID_0–8  hours_ was calculated as the weighted sum of the PIDs obtained from *t* = +1 hour (hr) to *t* = 8 hours (hr) on VAS using the following equation: SAPID = *∑*[PIDt × time (hr) elapsed since previous observation]. The secondary efficacy variables also included assessment for patient's clinical global impression for change in pain.

### 2.6. Tolerability Variables

Assessment of tolerability was done by recording patient's and physicians' global assessment on tolerability of the drug and proportion of the patients experiencing any drug-related adverse events.

### 2.7. Statistical Analysis

Assuming responder rate of 0.7 in control group, a sample size of 108 in each group had 80% power to detect an increase of 0.16 with a significance level (alpha) of 0.05 (two-tailed; GraphPad StatMate 2.00). Fischer's exact test was applied to observe if there are significant differences between the responder rates. The decreases in PI (VAS score), PID, and SAPID were calculated (Mean ± SD) for each group and compared between the groups by using unpaired *t-*test. The within-group comparison of VAS scores was done using paired *t-*test. Tolerability was assessed by evaluating the percentage of patients reporting side effect. Analysis of adverse events and global assessment of safety and efficacy was done using Fisher's exact test. For all statistical tests, a *P* value of less than 0.05 was considered significant.

## 3. Results

### 3.1. Patient Demography

Total 217 patients were recruited and completed the study of which 109 patients received DXD and 108 patients received DLD. The baseline demographic data for both groups were comparable (Table 1). The clinical diagnosis of acute renal colic was found to be consistent with the radiological diagnosis of renal calculus in about 65% patients in both groups.

### 3.2. Efficacy

**Figure 1:**
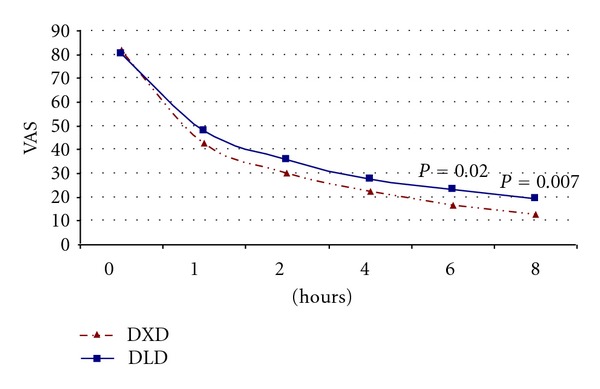
Improvements in VAS scores over 8 hours after DXD and DLD injections; unpaired *t*-test applied for between-group comparison.

The baseline VAS scores were comparable between the two groups (Table 1). There was a significant decrease in VAS scores as compared to baseline in both the groups starting from 1 hour after administration of the study drugs. The VAS score at the end of 6 hours and 8 hours was significantly less in DXD group as compared to DLD group (*P* = 0.02 for 6 hrs, *P* = 0.007 for 8 hrs) (Figure 1, Table 2).

There were significantly more responders (at least 50% improvement in VAS) in DXD group (98.17%) compared to DLD group (81.48%; *P* < 0.0001). The SAPID and PID at 8th hour were significantly more in DXD group compared to DLD group (*P* = 0.004 and 0.002) (Table 2).

**Figure 2:**
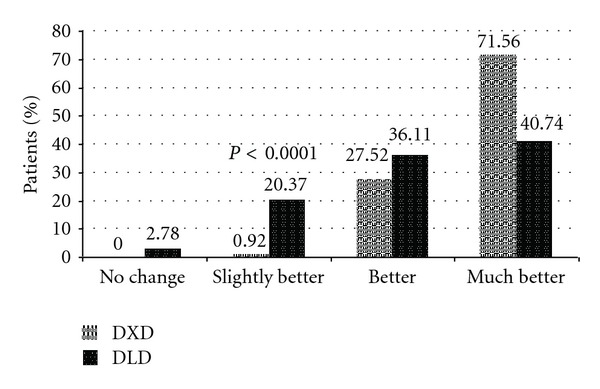
Patient-reported clinical global impression for change in pain. Fisher's exact test applied between proportion [(much worse + worse + slightly worse + no change) versus (slightly better + better + much better)].

In the patient-reported clinical global impression for change in pain, significantly more (71.56%) patients in DXD group demonstrated a “much better” response as compared to DLD group (40.74%, *P* < 0.0001). On the other hand, DLD group had more patients with “slightly better” response as compared to DLD group (20.37% versus 0.92, *P* < 0.0001). All patients in DXD group had improvement in pain, where as 2.78% patients in DLD group reported no change in pain (Figure 2).

### 3.3. Tolerability

The adverse events reported with DXD and DLD are depicted in Table 3. The incidence of vomiting and nausea occurred in relatively higher number of patients in DLD group. Incidence of all the other adverse events was comparable between the two groups.

**Figure 3:**
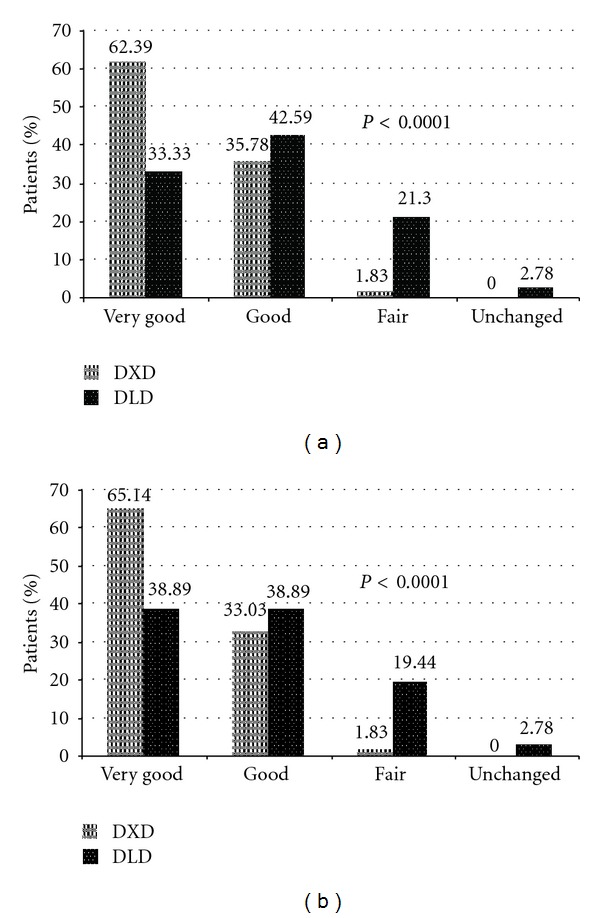
(a) Patient's global assessment of tolerability, (b) Physician's global assessment of tolerability. Fisher's extract test applied between proportions [(very good + good) versus (fair + unchanged)].

On patients' global assessment of tolerability, significantly more patients in DXD group rated the tolerability as good or very good (98.17% versus 75.92%; *P* < 0.0001) (Figure 3(a)). Similarly, 98.17% physicians reported favourable tolerability of DXD as compared to 77.78% for DLD (*P* < 0.0001) (Figure 3(b)).

## 4. Discussion

The analgesic and anti-inflammatory activity of ketoprofen is limited to its S(+)enantiomer or dexketoprofen and the R(−)enantiomer is devoid of any such activity. Use of dexketoprofen in place of ketoprofen offers distinct benefits such as same analgesic effect at lower doses, avoidance of excess metabolic load, and lack of adverse effects or drug interactions due to R-enantiomers [8, 9].

Oral dexketoprofen has been shown to have faster onset of analgesia than several other NSAIDs. Tromethamine salt of dexketoprofen is highly water soluble, which allows rapid and almost complete absorption of dexketoprofen [8]. Oral dexketoprofen is a first-line drug used for the treatment of mild-to-moderate acute pain and has shown its comparable efficacy as well as better tolerability than ketoprofen in several pain models such as dental pain, dysmenorrhea, and back pain [8, 10]. Parenteral administration of dexketoprofen has shown efficacy in reducing acute abdominal pain such as renal colic [3] and postoperative pain following hernia repair surgery [11]. Intramuscular dexketoprofen 50 mg was found to have faster, better, and longer analgesia than intramuscular diclofenac 50 mg [11].

NSAIDs are commonly used in clinical practice in combination with antispasmodics. Use of injectable NSAIDs and antispasmodics in ARC can subside the acute pain as well as reduce oedema and inflammation at the site of ureteric obstruction. It has been shown that addition of spasmolytic adds to the efficacy of NSAID in the treatment of acute spasmodic pain [6]. However, there are very few published studies assessing the safety and efficacy of such combinations and superiority of one combination over another. The results of the present study demonstrate that FDC of dexketoprofen and dicyclomine injection has better efficacy in reduction of ARC than FDC of diclofenac and dicyclomine injection, a commonly used FDC for acute spasmodic pain. The responder rate for DXD was more than 98% and the degree of analgesia achieved was significantly better than DLD. This was translated into significantly better patient-reported clinical global impression for change in pain. The results of this study were consistent with the results of a previous study on injectable dexketoprofen, which also showed better efficacy than diclofenac in the treatment of postoperative pain [11].

DXD was well tolerated as compared to DLD with more than 98% patients and physicians reporting good or very good tolerability for DXD as compared to 75–77% for DLD. DXD was also found to cause less incidence of vomiting than DLD. However, the total incidence of adverse events was comparable for DXD and DLD.

This study had a potential limitation that it was open-label, which could introduce bias. However, patients were not aware of the specific medication in the injection syringe, assuring unbiased response. In this study, we used diclofenac 50 mg instead of 75 mg as the commercially available FDCs of diclofenac with dicyclomine in the country contain no more than 50 mg of diclofenac.

## 5. Conclusion

This first report on the fixed-dose combination of dexketoprofen and dicyclomine injection shows that this product has superior efficacy and tolerability than the fixed-dose combination of diclofenac and dicyclomine injection in patients clinically diagnosed to be suffering from acute renal colic.

## Figures and Tables

**Table 1:** Demographic and baseline data.

	FDC of dexketoprofen and dicyclomine injection (DXD)	FDC of diclofenac and dicyclomine injection (DLD)	*P* value*
No of patients (*n*)	109	108	—

Age, years (Mean ± SD)	34.54 ± 10.87	36.86 ± 12.22	0.14

Sex (M : F)	79 : 30	68 : 40	0.15

Systolic BP, mm Hg (Mean ± SD)	126.53 ± 10.95	128.06 ± 11.58	0.32

Diastolic BP, mm Hg (Mean ± SD)	82.22 ± 7.40	81.89 ± 7.51	0.74

*Fisher's test applied for proportions and unpaired *t*-test for numerical data; SD: standard deviation.

**Table 2:** Efficacy parameters for DXD and DLD injections.

Variables	DXD (*n* = 109)	DLD (*n* = 108)	*P* value*
Responder rate (%)	98.17	81.48	<0.0001

Baseline VAS, (Mean ± SD)	81.97 ± 11.68	80.47 ± 12.44	0.36

VAS score at 8th hr, (Mean ± SD)	12.46 ± 15.18	19.35 ± 21.47	0.007

PID at 8th hr, (Mean ± SD)	69.51 ± 18.69	61.12 ± 20.00	0.002

SAPID, (Mean ± SD)	480.91 ± 156.67	420.35 ± 146.67	0.004

VAS: visual analogue scale, PID: pain intensity difference, SAPID: sum of pain intensity difference, *Fisher's test applied for proportions and unpaired *t*-test for numerical data, SD: standard deviation.

**Table 3:** Adverse events (ITT analysis).

Adverse event	(DXD); % (*n*) (*n* = 109)	(DLD); % (*n*) (*n* = 108)	*P* value*
Total no. of patients	11.93(13)	12.04 (13)	1.00
Burning micturition	2.75 (3)	0.93 (1)	0.62
Pain at injection site	2.75 (3)	0 (0)	0.25
Headache	1.83 (2)	0.93 (1)	1.00
Nausea	1.83 (2)	7.40(8)	0.06
Dryness of mouth	1.83 (2)	0 (0)	0.5
Generalised weakness	1.83 (2)	0.93 (1)	1.00
Giddiness	0.92 (1)	0.93 (1)	1.00
Weakness	0.92 (1)	0(0)	1.00
Cough	0 (0)	0.93 (1)	0.5
Vomiting	0 (0)	4.63 (5)	0.03

*Fisher's exact test ITT: Intention to treat.
